# Microbial meat substitutes: An annotated selection of World Wide Web sites relevant to the topics in *microbial biotechnology*

**DOI:** 10.1111/1751-7915.13610

**Published:** 2020-06-02

**Authors:** Lawrence P. Wackett

**Affiliations:** ^1^ Department of Biochemistry, Molecular Biology & Biophysics BioTechnology Institute University of Minnesota St Paul MN 55108 USA

## Meat substitute compilations

### https://www.sciencedirect.com/topics/food-science/meat-substitute

This site links to a number of papers on meat substitutes. Many deal with plant‐based products, but with some, microbial fermentations are part of the process.

## Air protein

### https://www.airprotein.com

The Air Protein company uses hydrogenotrophs to make bacterial cell mass from hydrogen and carbon dioxide. The cell mass is made into a protein powder that, in turn, can be formulated into meat‐like or paste‐like products.

## Nature’s fynd

### https://www.naturesfynd.com

The basis of Nature’s fynd’s technology is a protein from a fungus that was isolated from Yellowstone National Park. It is made into meatless burgers, dairy products and protein powders.

## Motif foodworks

### http://madewithmotif.com

Motif foods are based on animal‐free food products but include plant‐based, as well as microbe‐based, products.

## Microbes, Heme and Impossible burgers

### https://asm.org/Podcasts/MTM/Episodes/Microbes,-Heme,-and-Impossible-Burgers-with-Pat-Br

This link is to an American Society for Microbiology site that deals with establishment of the Impossible food company, one of the early entrants into the current rush for producing microbial materials to replace animal‐based foods.

## Edible microbes for sustainable food production

### https://link.springer.com/article/10.1007/s12571-019-00912-3

This review article highlights different approaches for using microbes more as food and discusses how this is more sustainable for society than traditional food sources.

## Microbial foods in a post‐plant world

### https://onezero.medium.com/microbe-scientists-are-preparing-us-to-eat-in-a-post-plant-world-245677693379

This article is more of a big picture treatment of the values of using microbes for foods, to highlight how lowering plant and animal agriculture is good for society and the environment.

## Meat substitutes from microorganisms

### https://www.gfi.org/animal-proteins-from-microorganisms

This page from the Good Food Institute contains a series of articles published 15 May 2019 or earlier. The emphasis is on microbial and plant‐based food products.

## Animal free meat from volcanic microorganisms

### https://www.gfi.org/blog-sustainable-bioproducts-volcanic-microorgan

This page from the Good Food Institute contains a series of articles published 30 October 2019 or earlier.

## Alternative meat is going to be $140 billion market

### https://www.weforum.org/agenda/2019/11/finnish-company-food-made-air-methane-microbes

This article focuses on the business aspects of microbe‐based food products and predicts that it will be a major growth market over the next decade.

## Microbial contributions to hamburgers

### https://www.asm.org/Articles/2019/May/The-Microbial-Reasons-Why-the-Impossible-Burger-Ta

This article goes into some of the technical aspects of the Impossible meatless hamburger, in which the red ‘meat’ colour is derived from microbially produced leghaemoglobin.

## Alternative protein

### https://singularityhub.com/2019/10/16/animals-are-out-alt-protein-is-in-and-its-cooking-up-some-unbelievable-creations/

This article highlights many of the big players in the commercial world of microbially produced foods and provides links to further information.

## Producing protein from methane

### https://www.bio.org/sites/default/files/legacy/bioorg/docs/0830AM-Lori%2520Giver.pdf

This link is to a slide deck from Calysta, a company using natural gas, methane, to produce microbe‐based animal and fish feed products.

## Microbial protein in a bubble‐free reactor

### https://backend.orbit.dtu.dk/ws/files/162261842/NRR18_SCP_final.pdf

This report describes a novel bioreactor with improved properties for cultivating methanotrophic bacteria for the production of single cell protein.

## Microbial protein for meatless meat

### https://sciencemediahub.eu/2019/12/18/is-microbial-protein-the-answer-to-the-quest-for-meatless-meat/

This page highlights research ongoing in Europe to develop food products based on microbially produced protein material.

